# Major Depressive Disorder Is Associated with Impaired Interoceptive Accuracy: A Systematic Review

**DOI:** 10.3390/brainsci9060131

**Published:** 2019-06-06

**Authors:** Michael Eggart, Andreas Lange, Martin J. Binser, Silvia Queri, Bruno Müller-Oerlinghausen

**Affiliations:** 1 Faculty Social Work, Health and Nursing, University of Applied Sciences Ravensburg-Weingarten, 88250 Weingarten, Germany; andreas.lange@hs-weingarten.de (A.L.); martin.binser@hs-weingarten.de (M.J.B.); silvia.queri@hs-weingarten.de (S.Q.); 2 Department of Psychiatry and Psychotherapy I, Ulm University, 88214 Ravensburg (Center for Psychiatry Südwürttemberg), Germany; 3 Drug Commission of the German Medical Association, 10623 Berlin, Germany; bruno.mueller-oerlinghausen@web.de; 4 Charité Universitätsmedizin Berlin, 10117 Berlin, Germany; 5 Brandenburg Medical School Theodor Fontane, 16816 Neuruppin, Germany

**Keywords:** major depressive disorder, interoception, heartbeat perception, somatic signals

## Abstract

Interoception is the sense of the physiological condition of the entire body. Impaired interoception has been associated with aberrant activity of the insula in major depressive disorder (MDD) during heartbeat perception tasks. Despite clinical relevance, studies investigating interoceptive impairments in MDD have never been reviewed systematically according to the guidelines of the PRISMA protocol, and therefore we collated studies that assessed accuracy in detecting heartbeat sensations (interoceptive accuracy, IAc) in MDD (databases: PubMed/Medline, PsycINFO, and PsycARTICLES). Out of 389 records, six studies met the inclusion criteria. The main findings suggest that (i) moderately depressed samples exhibit the largest interoceptive deficits as compared with healthy adults. (ii) difficulties in decision making and low affect intensity are correlated with low IAc, and (iii) IAc seems to normalize in severely depressed subjects. These associations may be confounded by sex, anxiety or panic disorder, and intake of selective serotonin reuptake inhibitors. Our findings have implications for the development of interoceptive treatments that might relieve MDD-related symptoms or prevent relapse in recurrent depression by targeting the interoceptive nervous system.

## 1. Introduction

The etiology and pathogenesis of major depressive disorder (MDD) symptomatology is complex and not sufficiently understood. Adult subjects regularly exhibit affective, cognitive, behavioral, psychomotor, and somatic symptoms [1]. The association between MDD and somatization is common and clinically highly relevant [2]. Individuals with MDD frequently suffer from pain, nausea, constipation, heart palpitations, shortness of breath, dizziness [2], or a common sense of physical malaise [3]. Meta-analytic evidence suggests an association between depression and functional somatic syndromes such as irritable bowel syndrome, non-ulcer dyspepsia, fibromyalgia, and chronic fatigue syndrome [4]. Moreover, functional somatic symptoms are positively correlated with depression severity [5,6]. Thus, there is clear evidence for clinically relevant associations between MDD and functional somatic symptoms that point to disturbed afferent bodily signals [7]. It has been shown that impaired interoception is associated with a variety of mental disorders [8]. This correlation between mental functions and the body is the core concept of embodiment theories [9,10] and might enable a deeper understanding of psychosomatic processes in MDD [11]. Therefore, this review focuses on the interoceptive system in order to shed new light on psychopathology and potential novel targets for the treatment of MDD. However, it is not in the scope of this review to determine if disturbed processing of afferent bodily signals is a cause or the result of mental disorders.

Interoception has been defined in various ways since Sherrington introduced the term in his seminal work [12,13]. In this review, we favor the broadly accepted definition by Craig [14] defining interoception as the sense of the physiological condition of the body. Accordingly, interoception comprises sensations from the entire body (e.g., thirst, hunger, itch, dyspnea, sexual arousal, heartbeat, distensions of bladder, stomach, rectum or esophagus, as well as temperature, pain, affective touch, etc.) and not just from the viscera. Craig’s definition is based on findings of functional neuroanatomy and relates to a sensory interoceptive pathway that conveys internal bodily signals to the brain where they are processed and integrated at the conscious or unconscious level [15]. The lamina I spinothalamocortical pathway constitutes the complementary afferent part to the efferent autonomic nervous system [14,16]. It originates in the small-diameter afferent fibers (Aδ and C) that carry sensory information about the physiological condition of virtually all bodily tissues. The sensory inputs are conveyed to the superficial dorsal horn of the spinal cord, where they are relayed to the brainstem. Ascending projections provide thalamocortical representations of the physiological condition of the body. Neuroimaging studies have shown that the activity of anterior insular cortex is correlated with awareness of the physiological condition (e.g., appetite during hypoglycemia) as well as bodily feelings [16,17]. Therefore, the insula has been referred to as the primary interoceptive cortex [16]. Afferent bodily signals serve as a basis for several physiological and psychological functions. Particularly, interoception and the related lamina I spinothalamocortical pathway are closely associated with homeostasis. As a result, interoception motivates behaviors that face homeostatic needs [18]. Furthermore, interoception is closely associated with emotions, as well as cognitive functions [18,19]. It has been suggested that unspecific states like subjective well-being are built upon an integration and evaluation of interoceptive signals that are represented in the insula and associated structures [14,20]. Additionally, active Bayesian inference models of interoception hold that interoceptive experience is shaped by brain predictions which are based on prior beliefs and expectations about bodily states. Prediction errors, defined as a mismatch between afferent inputs and brain’s predictions, are suspected to underlie various mental disorders, e.g., anxiety and depression [21]. The relevance of predictive coding models for psychiatry has been discussed in detail elsewhere [15,22,23,24].

Garfinkel et al. proposed three different dimensions of interoception which have been quantified with distinct methodological paradigms [25,26]. This review focuses on interoceptive accuracy (IAc), also referred to as interoceptive sensitivity, which is defined as the “objective accuracy in detecting internal bodily sensations” [26] (p. 67). Other facets of interoception comprise interoceptive sensibility (“self-perceived dispositional tendency to be internally self-focused and interoceptively cognisant”, p. 67) and a metacognitive dimension of interoception. IAc is commonly assessed using heartbeat tracking [27] or heartbeat discrimination tasks [28]. Schandry’s heartbeat tracking method has been preferred in psychiatric research due to its ease of use. Briefly, the task involves the following: A researcher asks individuals to count the number of their heartbeats by focusing on physical sensations in repeated trials at different time intervals. Reported number of heartbeats in each trial are compared to the objectively assessed number of heartbeats recorded by electrocardiography. An error index is calculated and averaged across trials that represents an outcome measure of the heartbeat perception accuracy for each participant [27].

Several neuroimaging studies investigated brain function during the heartbeat perception task (for a meta-analysis, see [29]). For example, Critchley et al. [20] demonstrated that an activation of the right anterior insular cortex predicted higher accuracy on a heartbeat perception task. Respectively, the authors showed that grey matter volume of the right anterior insular cortex predicted higher IAc in healthy adults. However, it has been shown that insula activity negatively correlates with depression and somatic symptom severity during a heartbeat perception task in subjects with MDD [30]. Taking these findings together, current neuroscientific knowledge suggests an involvement of the insula on heartbeat perception with MDD subjects showing hypoactivation of the insular cortex.

In summary, we will draw two conclusions: First, the hypoactivation of the insula in depressed individuals during a heartbeat perception task suggests that MDD is associated with altered interoceptive awareness of the cardiovascular system. Second, the complex psychosomatic symptomatology of MDD points to disturbed interoceptive processing [31,32,33]. There is preliminary evidence suggesting that MDD is associated with altered IAc during a heartbeat perception task [21,34]. However, previous literature reviews have been subject to main limitations since they were narrative or lacked a systematic search strategy according to recommendations of the PRISMA statement [35]. Thus, it is unclear if available reviews have covered the present state of research. Therefore, we systematically reviewed studies that assessed performance on heartbeat perception tasks in subjects with MDD. The main research questions of this systematic review were:

1. Do differences exist between subjects with MDD and other clinical samples as well as healthy control groups regarding accuracy on a heartbeat perception task?

2. Which type, and strength of association, exists between depression severity and performance scores on a heartbeat perception task?

We further aimed to explore associations between MDD-related symptoms and interoception as well as interoception altering effects of antidepressants in retrieved studies to identify possible confounding effects.

## 2. Methods

### 2.1. Search Strategy

The literature search was conducted in the electronic databases PubMed/Medline, PsycINFO, and PsycARTICLES. The database search was carried out in May 2017. There was no limit set regarding the publication year.

The search strategy is exemplified for PubMed/Medline. The search was conducted by means of medical subject headings (MeSH) and truncations. The search terms were combined with Boolean operators as follows: (“Depression”[MeSH] OR “Depressive Disorder”[MeSH] OR “Depressive Disorder, Major”[MeSH]) AND (interocept* OR heartbeat* OR “heartbeat detection” OR “heartbeat perception” OR “heart beat perception” OR “heartbeat awareness” OR “heartbeat tracking” OR “heartbeat discrimination” OR “cardiac awareness” OR “cardiac sensitivity” OR “cardiac perception”). Additionally, further papers were identified in reference lists of retrieved articles and screened for eligibility (backward snowballing).

### 2.2. Eligibility Criteria

Peer reviewed articles were included that met the following inclusion criteria: (a) studies (with or without control group) that investigated the association between MDD and IAc; (b) existence of a reliable clinical diagnosis of MDD (psychiatric or somatic comorbidity was accepted); and (c) assessment of IAc as a primary or secondary outcome measure gauged by the heartbeat tracking [27] or the heartbeat discrimination task [28], both subsumed under the term heartbeat perception task. In terms of publication status, articles in print or published ahead of print were accepted. The exclusion criteria were: (a) comorbid depressive symptoms without MDD diagnosis, (b) no assessment of IAc, (c) literature reviews, or (d) no available full text.

### 2.3. Study Selection

Each study was inspected for eligibility by the first author after reading the title and abstract. This process was followed by a full-text review of potentially relevant studies. Any uncertainties regarding eligibility criteria were discussed and resolved among all the authors. Each decision for inclusion or exclusion was summarized in a flow chart according to the PRISMA recommendations [35].

### 2.4. Data Extraction

The following data was extracted from eligible studies and included in the review: depression diagnostic criteria, basic participant characteristics, type of comparison groups, depression severity scores, participant’s exclusion criteria, heartbeat perception scores (IAc respectively error rates) and type of heartbeat perception task. Moreover, main findings of included studies were summarized in a qualitative synthesis.

## 3. Results

### 3.1. Process of Study Selection

**Figure 1:**
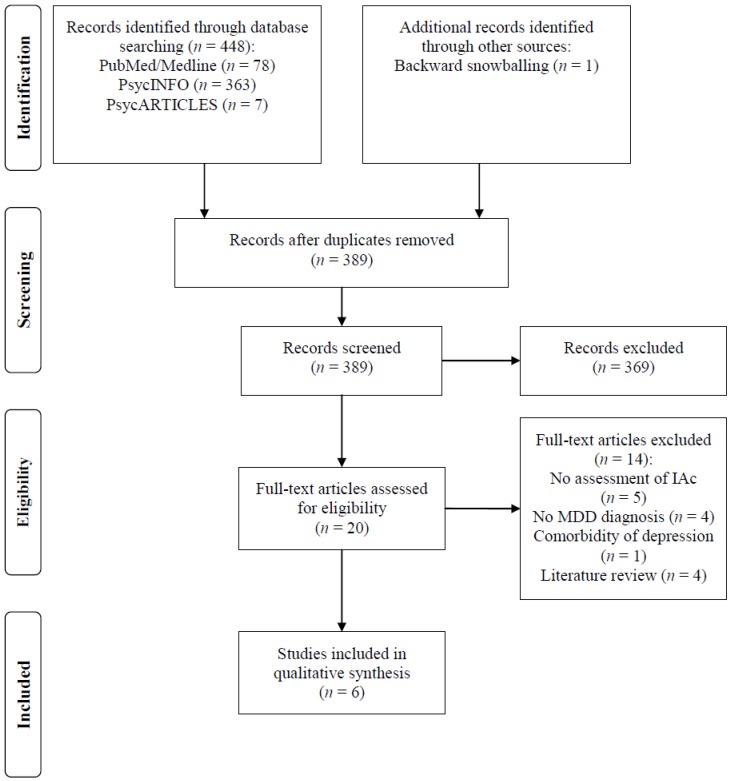
PRISMA flowchart of study selection process.

A flow diagram outlines the study selection process (Figure 1).

The electronic database search retrieved 448 publications (Figure 1). Backward snowballing additionally identified one eligible article. After removing duplicates, 389 studies remained. Twenty articles were identified as potentially relevant after screening of titles and abstracts. These studies were assessed for eligibility in full text. Of these, six studies met the eligibility criteria and were included in the review. The main reasons for study exclusion were: a lack of reported outcome measure on a heartbeat perception task, assessment of interoceptive dimensions other than IAc (e.g., interoceptive sensibility), absence of a reliable clinical diagnosis of MDD, or comorbidity of depression with a principal psychiatric diagnosis other than MDD (e.g., panic disorder).

### 3.2. Characteristics of Included Studies

Key characteristics of included studies are summarized in Table 1.

All retrieved studies were cross-sectional and published between 1992 and 2013. Study participants were recruited as inpatients or outpatients. To the best of our knowledge, IAc has not been studied in children or adolescents who suffer from depression. One study intended to control for sex by solely recruiting women [41]. A diagnosis of depression was based upon structured clinical interviews according to DSM-III-R, DSM-IV, or ICD-9 criteria. Five studies defined eligibility criteria for participant exclusion. The main reasons for exclusion were as follows: a diagnosis of MDD with comorbid panic or anxiety disorder, pregnancy, endocrine or cardiovascular disorder, cardiac medication, history of psychosis or substance abuse, brain injury, learning disability, and impaired cognitive ability. One observational study did not define any eligibility criteria [38]. All included studies used the heartbeat mental tracking task by Schandry [27] to assess IAc. Half of the studies explicitly intended to compare interoceptive functioning in MDD with healthy controls [39,40,41]. Other studies compared mood disorder samples with panic and anxiety disorder [36,37] or with other diagnostic groups [38]. Additionally, two studies estimated the type and strength of association between depression severity and IAc [39,41].

In the following sections, we will first summarize main findings of group comparisons. Second, we will review the type and strength of association between IAc and depression severity. Third, we will review possible confounding effects of modern antidepressants on IAc assessment. Fourth, we will report associations between IAc and MDD-related symptoms.

### 3.3. Group Differences on Heartbeat Perception Task Performance

Ehlers et al. [36] recruited a mixed sample of subjects with MDD and dysthymia (Beck Depression Inventory (BDI): *M* = 17.50, *SD* = 11.20; Note: To establish accordance with APA style requiring that statistics should be rounded to two decimal places, we added a zero to statistics if cited authors reported results that were rounded to one decimal place.) who exhibited significantly lower ability to accurately estimate their heartbeats than subjects with panic (BDI: *M* = 11.50, *SD* = 5.50) or generalized anxiety disorder (BDI: *M* = 15.30, *SD* = 11.80). In an extended replication study, a comparable mood disorder sample displayed worse performance on a heartbeat mental tracking task than subjects with panic disorder, even though group differences did not yield statistical significance (BDI scores not reported). However, post-hoc analysis revealed a significantly lower proportion of accurate heartbeat perceivers in the mood disorder sample as compared with the panic disorder group [37]. The study by Mussgay et al. [38] found a nonsignificant trend for decreased IAc in reactive depression as compared with healthy controls (BDI scores not reported). However, there were no significant IAc differences between subjects with affective and panic disorder.

Study designs with improved quality have been established since 2007. Dunn et al. [39] reported that a moderately depressed community sample (BDI: *M* = 22.20, *SD* = 8.10) exhibited lower IAc than healthy controls (BDI: *M* = 4.30, *SD* = 3.00). However, a severely depressed in- and outpatient sample (BDI: *M* = 28.30, *SD* = 9.00) displayed significantly better IAc than the moderately depressed community volunteer sample (IAc and error scores are reported in Table 1). Even though the in- and outpatient MDD sample also exhibited lower IAc than healthy controls, there were no statistically significant group differences. These results remained unchanged after controlling for state and trait anxiety. In contrast, Terhaar et al. [40] found that MDD subjects (BDI: *M* = 22.63, *SD* = 10.16) showed significantly less accuracy on a heartbeat perception task than healthy matched controls (BDI: *M* = 2.31, *SD* = 2.88). However, depression severity in the sample by Terhaar et al. [40] was equivalent to the moderately depressed community sample by Dunn et al. [39]. The effect of group was large in the study by Terhaar et al. (Cohen’s *d* = 0.80). A significant group difference between the MDD and healthy controls has also been found by Furman et al. [41] where the MDD females without comorbid anxiety disorder (BDI-II: *M* = 25.00, *SD* = 9.20) exhibited significantly lower IAc than healthy controls (BDI-II: *M* = 1.90, *SD* = 3.10, Cohen’s *d* = 0.49). Taking these findings together, group differences between individuals with MDD and healthy controls depend on depression severity and are potentially confounded by anxiety scores and sex [41]

### 3.4. Association between Interoceptive Accuracy and Depression Severity

Few papers investigated the association between IAc and BDI scores in correlational analysis. Dunn et al. [39] found that higher BDI scores were associated with lower performance on a heartbeat mental tracking task (*r* = −0.53, *p* = 0.03) in a sample of moderately depressed community volunteers. In contrast, this correlation disappeared in the severely depressed in-and outpatient sample. Comparable results were reported by Furman et al. [41] who found that depression severity was uncorrelated with IAc in MDD outpatients. However, there was a trend towards a negative correlation between BDI scores and IAc in healthy controls. Given the empirical evidence that a moderately depressed sample displayed worse performance on a heartbeat perception task than a severely depressed in- and outpatient sample (Cohen’s *d* = 1.29) as well as healthy controls (Cohen’s *d* = 0.85), Dunn et al. [39] tested the association between BDI and IAc scores in a nonlinear regression model. The authors showed that a quadratic regression model yielded a model fit of *R*^2^ = 0.13 across a pooled sample of all study participants. Therefore, Dunn et al. suggested an inverted U-shaped relationship between the performance in a heartbeat mental tracking task (i.e., IAc) and the BDI scores across healthy to moderately to severely depressed participants.

### 3.5. Modern Antidepressants as Possible Confounders

Five of the included studies explored possible confounding effects of drugs on accuracy on a heartbeat mental tracking task performance. Ehlers et al. [36] examined if the difference in IAc scores between the panic disorder and depression group disappears after taking into account medication status (Note: The paper by Ehlers et al. [36] did not report the participant’s medication status in detail. However, the authors mentioned that subjects were included in the study who were treated with benzodiazepines, whereas, subjects with cardiovascular medication were excluded.). However, significant group differences remained after exclusion of medicated subjects, pointing to higher IAc in the panic disorder sample independent of medication status (sample sizes *N* were not reported). Mussgay et al. [38] compared a sample of heterogeneously medicated (cardiac medication, antidepressants, analgesics, antidiabetics, etc.) individuals (*N* = 229) with unmedicated subjects (*N* = 188), both diagnosed with neurotic depression. There were no significant differences for heartbeat perception scores found between these groups. Accordingly, Furman et al. [41] did not find any significant accuracy score differences between individuals with MDD who were medicated (*N* = 15) or not medicated (*N* = 10) with psychoactive drugs. However, the authors did not report on specified drug groups or single active ingredients.

Considering modern antidepressants, two studies explicitly explored if selective serotonin reuptake inhibitors (SSRIs), or serotonin-norepinephrine reuptake inhibitors (SNRIs) alter heartbeat perception in MDD. In a study by Dunn et al. [39], all participants in the severely depressed sample were treated with SSRIs alone (*N* = 14) or in combination with anxiolytic or soporific drugs (*N* = 4). The medication status of the community depressed (*N* = 18) and the control sample (*N* = 18) were not reported. After collapsing the three samples and splitting them into medicated versus unmedicated groups, a comparison of IAc scores yielded significant group differences after controlling for depression severity. In detail, medicated subjects performed significantly better on a heartbeat mental tracking task than unmedicated subjects. Thus, it has been suggested that SSRI intake is associated with more accurate heartbeat perception (i.e., SSRIs possibly exert an IAc increasing effect). In contrast, Terhaar et al. [40] found no significant group differences between subjects mainly taking SNRIs (73% SNRIs, 18% SSRIs) and antipsychotics (9% Aripiprazol or Olanzapine, respectively) versus unmedicated subjects diagnosed with MDD. However, sample sizes were too low (medicated: *N* = 11, unmedicated: *N* = 5) in terms of statistical hypothesis testing. In addition, the authors did not report descriptive statistics for post-hoc trend analysis.

### 3.6. Associations between Interoceptive Accuracy and MDD-Related Symptoms

Furman et al. [41] have found that IAc is associated with specific MDD-related symptoms. The intensity of the positive affect is significantly correlated with performance on a heartbeat perception task (*r* = 0.42, *p* = 0.04). Thus, a lower ability to accurately estimate the number of one’s heartbeat has shown to be more prevalent in participants who exhibited reduced intensity of positive emotions. In addition, the authors investigated the association between indecisiveness and performance on Schandry’s heartbeat mental tracking task. A binary logistic regression model revealed that higher error scores on a heartbeat mental tracking task significantly predicted difficulties in decision making. This association remained statistically significant after controlling for depression and anxiety severity scores (*β* = 5.95, *Wald χ*^2^(1) = 4.30, *p* = 0.04). A further analysis revealed a significant group effect between study participants with: (a) MDD and decision-making difficulties, (b) MDD without decision-making difficulties and (c) healthy controls. The mean heartbeat perception accuracy scores were significantly lower in individuals with MDD who exhibited decision-making difficulties than in individuals with MDD without decision-making difficulties.

## 4. Discussion

This systematic review collated studies that assessed performance on a heartbeat perception task in individuals with MDD. Our results are consistent with previous narrative reviews which reported heartbeat-related interoceptive impairments in MDD [8,21,34]. The main findings suggest that differences between subjects with MDD and healthy controls on a heartbeat mental tracking task performance depend on depression severity. Moderately depressed subjects displayed significantly lower performance in accurately estimating their heartbeats than healthy controls. However, current evidence suggests that IAc normalizes with increasing depression severity from moderate to severe depression with moderate depressed subjects exhibiting the greatest interoceptive deficits. For example, such a normalizing effect may explain why the more severely depressed in- and outpatient sample in a study by Dunn et al. did not significantly differ from the healthy controls with respect to their accuracy of heartbeat perception. In contrast, the moderately depressed sample in a study by Terhaar et al. displayed significantly lower IAc than the healthy matched controls. These findings also indicate that the association between depression severity and performance scores on a heartbeat perception task (i.e., IAc) is nonlinear. While no significant linear correlation between IAc and depression severity has been found in MDD samples [39,41], a significantly negative and large linear correlation has been found in a moderately depressed community volunteer sample [39]. Taking all findings of retrieved studies together, preliminary evidence suggests an inverted U-shaped curvilinear relationship between BDI scores and error rates on heartbeat mental tracking trials. This means that IAc scores are currently best modeled as a quadratic function of depression severity illustrated by a parabola opening downwards (inverted U). From a clinical point of view and according to preliminary evidence, interoceptive impairments are mostly marked in moderately depressed subjects implying that the cortical representations of cardiac activity are most attenuated in these patients.

### 4.1. Preliminary Explanations for the Inverted U-Shaped Relationship

Various explanations have been discussed that aimed to explain the inverted U-shaped relationship [34]. In summary, two explanatory approaches are prominent in literature. First, a nonlinear main effect of depression severity on IAc has been speculated by Dunn et al. [39]. Second, some authors argued that various factors might confound the association between depression severity and IAc. Against the background of this review, we will discuss these explanations and will draw several conclusions.

Previous research has shown that depression and anxiety have opposite effects on IAc [21,42]. Consequently, comorbid anxiety or panic disorder could explain the restoration of IAc from moderate to severe depression, since anxiety or panic symptoms are correlated with increasing depression severity [43]. Thus, Furman et al. [41] recruited female participants without comorbid anxiety disorder in order to control for confounding effects. Study participants had higher depression severity scores than in the study by Terhaar et al., but lower than in the study by Dunn et al. Even though women are more likely to perform less accurately on a heartbeat perception task than men [44], the sample exhibited significantly lower IAc than the healthy matched controls. This might support the hypothesis that anxiety or panic symptoms exert a restorative function on interoception. However, the study by Dunn et al. showed that IAc scores of the severely depressed in- and outpatient sample did not significantly differ from the healthy controls even after covarying out state and trait anxiety. Hence, it is currently unclear whether a confounding effect of anxiety or panic symptoms exists that might explain the nonlinear association between depression severity scores and IAc. Instead, as already reasoned by Dunn et al. [39], depression severity might exert its own nonlinear main effect on IAc. Beyond a main effect of depression severity, an interaction effect of antidepressant medication status could also be assumed as a possible confounder of the association. Preliminary evidence tentatively points to the SSRI group because SSRI intake might be correlated with increased cardiovascular IAc [39]. This might also explain why severely depressed samples exhibit normalized IAc scores, since SSRI intake is common in severely depressed individuals (in the Dunn et al. study, for example, all of the severely depressed individuals were treated with SSRIs). We hypothesize that intake of SSRIs might increase IAc for several reasons. These drugs possess clearly excitatory effects resulting frequently in sleeplessness, agitation, anxiety, palpitations, psychomotor unrest, and even provocation of suicidal ideation and suicidal behavior in rare cases [45]. One could speculate that these principally adverse drug reactions might also result into increased attentiveness to interoceptive signals. Moreover, SSRI intake is associated with a dose-dependent corrected QT interval prolongation [46] which could alter IAc. In summary, there is current uncertainty whether antidepressant medication status, depression severity alone, or an interaction effect between both explain the normalization of IAc in severely depressed individuals.

### 4.2. Interoception and Its Link to Affective and Cognitive Disturbances in MDD

There is evidence that reduced intensity of emotional experience is correlated with impaired interoceptive functioning in depression [41]. Psychological research has shown that MDD alters emotional reactivity by attenuating both positive and negative emotions [47]. The findings are consistent with so-called arousal theories of emotion [48,49,50,51], as well as with the embodiment paradigm [9]. These concepts emphasize the body and related physiological states as crucial for emotional experience and cognition. As already proposed by William James [48] and later supported by Schachter and Singer [49], physiological arousal is a determining factor for the development of emotional states. In this context, it is noteworthy to mention that already James [48] stated ahead of his time, “A purely disembodied human emotion is a nonentity. I do not say that it is a contradiction in the nature of things, or that pure spirits are necessarily condemned to cold intellectual lives; but I say that for *us*, emotion dissociated from all bodily feelings is inconceivable” (p. 194). Accordingly, it has been proposed that interoception serves as a key mechanism of embodiment by continually representing afferent bodily signals that accompany emotional states [10]. A state of disembodiment in the form of self-objectification has been observed in adults who were classified as poor heartbeat perceivers [52]. Some authors reasoned that impaired interoception is associated with higher levels of self-harm and suicidality with suicide attempters exhibiting worse interoception than nonattempters [53]. Additionally, persons on the upper end of the suicidality continuum are more prone to exhibit interoceptive impairments [54]. Therefore, mitigation of these disembodied states may be a promising strategy to prevent suicidal behavior [53].

Furthermore, there is evidence for a negative correlation between MDD-related indecisiveness and IAc [41]. These findings are consistent with Damasio’s somatic marker hypothesis holding that differentiable bodily feelings or “homeostatic emotions” [55] are influencing and particularly guiding the process of decision making [50,51]. Thus, MDD-specific indecisiveness could be attributed to these interoceptive impairments since bodily feedbacks from the cardiovascular system are related to the process of intuitive decision making [56].

### 4.3. Limitations

This review is subject to various limitations. First, the design of the included studies varied considerably with respect to recruitment strategies, subject’s depression severity, exclusion criteria, comparison groups, and covariate control (e.g., anxiety or panic symptoms, medication status). Due to this heterogeneity, we focused on summarizing the main findings of the included studies in a qualitative synthesis rather than in a meta-analysis. Second, no study could be retrieved that used the heartbeat discrimination task [28] to assess IAc. Schandry’s heartbeat mental tracking and Whitehead’s discrimination task assess different aspects of IAc [26]. Third, Schandry’s heartbeat mental tracking task itself is subject to certain limitations. It has been demonstrated that heartbeat perception is trainable by the task itself [57]. Another problem is the non-standardized use of instructions as well as concerns about validity and reliability of the task [58]. Fourth, the included studies that investigated the effect of medication status on IAc, other than SSRI, were essentially underpowered due to small sample sizes and potentially biased with regard to heterogeneous drug groups. Fifth, this review only focused on IAc of the cardiovascular system rather than other organ-specific modalities. However, studies have shown that performance on a heartbeat mental tracking task positively correlates with sensitivity for gastrointestinal stimuli [59,60]. Hence, a generalization of our results across interoceptive impairments in other organ systems seems justified, except for the respiratory system [58].

### 4.4. Clinical Implications

Interoception has been linked with homeostatic control, affect and emotion perception, as well as emotion regulation, social cognition, motivation, memory, decision making, self-awareness, time perception, sexual pleasure, and well-being [14,16,18,19,61]. Afferent sensory inputs from within the body are considered as crucial for understanding psychopathology [32,62] and psychosomatic processes [31]. Accordingly, disturbed interoception might be causally related to affective and somatic symptoms in MDD (for a review, see [34]). Furthermore, interoception has been discussed as a possible new biomarker in psychiatry [8]. Despite accumulating evidence of impaired interoception in several mental disorders (MDD, anxiety and panic disorder, autism spectrum disorder, somatic symptom disorder, and eating disorders) [7,21,63,64,65], the use of therapies that target the interoceptive system is scarce in clinical practice [15] and has only been established for panic disorder in the form of interoceptive exposure [8]. There is evidence that mindfulness-based cognitive therapy (MBCT) activates interoceptive networks in subjects at high familial risk for bipolar disorder [66]. However, it is unclear if MBCT’s relapse preventing effects in recurrent MDD [67] are mediated by restoration of impaired interoceptive functioning. Furthermore, an interoceptive mechanism of action could also be assumed for the antidepressant effects of whole-body hyperthermia [68]. It will be important for future research to develop and test interoceptive treatments for subjects with MDD that may have the potential to prevent or relieve depression-related symptoms or suicidal lethality. In this context, particular massage interventions which have recently been linked with an interoceptive mechanism of action are considered as promising approaches [69].

### 4.5. Future Research Directions

We propose to thoroughly test the inverted U-shaped relationship in future studies. These studies should also clarify if the nonlinear association possibly stems from an experimental artefact based on the influence of confounding factors. Validity of Schandry’s heartbeat mental tracking task is indeed affected by several physiological and psychological variables that are described elsewhere [58,70]. Besides, it is indispensable for future research to test the confounding risk of psychoactive drugs (e.g., SSRI) on performance on a heartbeat mental tracking task [39]. We propose to compare severely depressed and medicated subjects with severely depressed drug-naïve individuals. Furthermore, dose and duration of psychoactive or other drug treatment and their possible consequences on interoception should be taken into consideration.

Other future research directions imply a differentiated assessment of interoception across all dimensions (i.e., interoceptive sensibility, interoceptive accuracy, and interoceptive awareness [26]) as well as their interrelations in MDD. In addition, association between interoception and MDD-specific symptoms (rumination, anhedonia, fatigue, decrease/increase of appetite, insomnia, etc.) are understudied yet. For example, it has been shown that aberrant activity of the insula is related to functional somatic symptom severity in MDD [30]. However, performance scores on a heartbeat mental tracking task and other interoceptive outcome measures have never been correlated with somatic symptom severity in subjects with MDD, except for disturbances of sleep [71]. Moreover, possible associations between disturbed subjective time perception in MDD (for a meta-analysis, see [72]) and IAc have never been investigated, even though time perception has been correlated with activation of the anterior insular cortex and has been closely linked to interoception [73,74,75]. Furthermore, longitudinal studies are needed to investigate interoceptive impairment and its potential role as a significant predictor of early onset and adverse course of depression. Finally, future research should examine if impaired interoception is causally involved in the etiology, pathogenesis, and maintenance of MDD.

## 5. Conclusions

Can individuals who suffer from MDD perceive and accurately count the number of their heartbeats? Summarizing current evidence, the answer depends on depression severity. While moderately depressed individuals are poor heartbeat perceivers compared with healthy adults, these interoceptive impairments may be restored in severely depressed subjects. Due to the limited number of published studies, uncertainty exists as to whether the normalization of IAc in severe depression stems exclusively from a main effect of depression severity, or stems from a confounding effect (e.g., psychotropic drug intake or comorbid anxiety/panic). Clarification of these issues imply high impact for future research since impaired interoception might play a substantial role in the pathogenesis and maintenance of MDD symptomatology. Evidence suggests that low accuracy of heartbeat perception is linked with affective, cognitive and somatic symptoms of depression and is also associated with abnormal interoceptive representations of heartbeat sensations within the insula. Thus, considering clinical implications, experimental approaches targeting the interoceptive nervous system could possibly broaden current antidepressant treatment options.

## Figures and Tables

**Table 1:** Characteristics of included studies.

Study	Diagnostic Criteria	Participants and Comparison Groups (*N*, % Female, Age: M ± SD)	Exclusion Criteria	Heartbeat Perception Task (Number of Trials)	Main Findings (IAc Scores Resp. Error Rates in % Reported)
Ehlers and Breuer (1992) [36]	DSM-III-R	MDD or dysthymia (combined *N* = 16, 47% female, age: 36.80 ± 7.30 years) vs. panic disorder (*N* = 13, 38% female, age: 41.00 ± 6.80 years) vs. generalized anxiety disorder (*N* = 15, 44% female, age: 40.50 ± 9.50 years)	Pregnancy, endocrine or cardiovascular disorder, cardiac medication	HMTT (3 trials)	Depressed subjects exhibited significantly higher error for heartbeat perception (43.00%) than subjects with panic (23.00%) or generalized anxiety disorder (21.00%). Group differences remained significant after controlling for medication status.
van der Does et al. (1997) [37]	DSM-IV	MDD or dysthymia (combined *N* = 16, 81% female, age: 40.40 ± 13.00 years) vs. panic disorder (*N* = 23, 65% female, age: 41.10 ± 10.60 years) vs. healthy controls (*N* = 21, 62% female, age: 44.50 ± 14.10 years)	Lifetime history of psychosis or substance abuse, comorbidity of panic disorder	HMTT (3 trials)	No significant IAc difference between individuals with mood disorder (42.70%), panic disorder (30.30%), and healthy controls (37.10%). However, a significant lower proportion of accurate heartbeat perceivers was found in the mood disorder group (0.00%) as compared with subjects with panic disorder (30.40%), but no significant differences with regard to healthy controls (9.50%).
Mussgay et al. (1999) [38]	ICD-9	Neurotic depression (*N* = 141, 87% female, age: 41.10 years ± NA) vs. depressive psychogenic reactions (*N* = 106, 77% female, age: 42.80 years ± NA) vs. functional disorder (*N* = 43, 79% female, age: 38.90 years ± NA) vs. functional cardiac disorder (*N* = 48, 58% female, age: 45.10 years ± NA) vs. personality disorder (*N* = 26, 69% female, age: 35.40 years ± NA) vs. panic disorder (*N* = 53, 58% female, age: 41.30 years ± NA) vs. healthy controls (*N* = 48, 60% female, age: 35.80 years ± NA)	No exclusion criteria (observational study that recruited all inpatients in the sequence of their admission)	HMTT (3 trials)	The groups significantly differed regarding performance in HMTT. There was a post-hoc trend (*p* = 0.06) towards lower IAc in reactive depression (0.45) as compared with healthy controls (0.56). No IAc score difference between medicated and unmedicated subjects (IAc scores not reported), except for panic disorder: medicated (0.30) vs. unmedicated (0.57).
Dunn et al. (2007) [39]	DSM-IV	MDD in-/outpatients (*N* = 18, 72% female, age: 47.10 ± 9.90 years) vs. moderately depressed community volunteers (*N* = 18, 72% female, age: 40.10 ± 15.60 years) vs. healthy controls (*N* = 18, 78% female, age: 44.80 ± 13.00 years)	Comorbidity of panic disorder, no history of brain injury, psychosis, learning disability or substance abuse	HMTT (6 trials)	The moderately depressed community sample exhibited lower heartbeat perception accuracy (39.60%) than healthy controls (29.00%). No significant difference in IAc between subjects with MDD (25.31%) and healthy controls. Significant negative correlation between IAc and depression severity in community volunteers, *r* = −0.53, *p* = 0.03. No significant correlation between IAc and depression severity in subjects with MDD. A curvilinear inverted U-shaped relationship was found between depression severity and IAc (error rate) across all participants (*R*^2^ = 0.13). Higher IAc in medicated subjects (mainly SSRI) than in unmedicated individuals after collapsing the three samples (adjusted error rates not reported).
Terhaar et al. (2012) [40]	DSM-IV	MDD (*N* = 16, 81% female, age: 21.75 ± 12.40 years) vs. healthy matched controls (*N* = 16, 75% female, age: 39.81 ± 17.60 years)	Comorbidity of panic disorder	HMTT (3 trials)	Participants with MDD exhibited significantly lower IAc (0.67) than healthy controls (0.81, large effect, Cohen’s *d* = 0.85). No significant IAc difference between medicated and unmedicated subjects (IAc scores not reported).
Furman et al. (2013) [41]	DSM-IV-TR	MDD without anxiety disorder (*N* = 25, 100% female, age: 38.20 ± 11.90 years) vs. healthy controls (*N* = 36, 100% female, age: 36.00 ± 12.50 years)	Psychosis, substance abuse or impaired mental status, cardiovascular symptoms	HMTT (3 trials)	IAc was significantly lower in subjects with MDD (0.55) than in healthy controls (0.65). Depression severity and IAc were uncorrelated in MDD as well as in healthy controls. IAc was positively correlated with positive affectivity in MDD. Low IAc was a predictor of indecisiveness, i.e., difficulties in decision making were more severe for inaccurate heartbeat perceivers (0.47) diagnosed with MDD than in subjects without decision-making difficulties (0.67). No significant IAc difference between individuals under psychoactive medication and unmedicated participants (no IAc scores reported).

DSM-III-R = Diagnostic and Statistical Manual of Mental Disorders, third edition, revised; DSM-IV(-TR) = Diagnostic and Statistical Manual of Mental Disorders, fourth edition (text revision); HMTT = Assessment of IAc with Schandry’s heartbeat mental tracking task (Schandry, 1981); IAc = Interoceptive Accuracy; ICD-9 = International Statistical Classification of Diseases and Related Health Problems, ninth revision; MDD = major depressive disorder; *N* = sample size; NA = data not available; *M* ± *SD* = mean and standard deviation. To establish accordance with APA style requiring that statistics should be rounded to two decimal places, we added a zero to statistics if cited authors reported results that were rounded to one decimal place.
